# Pathogen group-specific risk factors for intramammary infection in water buffalo

**DOI:** 10.1371/journal.pone.0299929

**Published:** 2024-04-04

**Authors:** Shuvo Singha, Gerrit Koop, Md. Mizanur Rahman, Fabrizio Ceciliani, Maria Filippa Addis, Md. Matiar Rahman Howlader, Mohammed Kawser Hossain, Renata Piccinini, Clara Locatelli, Ylva Persson, Valerio Bronzo

**Affiliations:** 1 Department of Veterinary Medicine and Animal Sciences, Università degli Studi di Milano, Lodi, Italy; 2 Laboratorio di Malattie Infettive degli Animali (MiLab), Università degli Studi di Milano, Lodi, Italy; 3 Department of Physiology, Veterinary, Animal and Biomedical Sciences, Sylhet Agricultural University, Sylhet, Bangladesh; 4 Department of Medicine and Surgery, Chattogram Veterinary and Animal Sciences University, Chattogram, Bangladesh; 5 Udder Health Bangladesh, Chattogram, Bangladesh; 6 Department of Population Health Sciences, Sustainable Ruminant Health, Faculty of Veterinary Medicine, Utrecht University, Utrecht, The Netherlands; 7 Department of Surgery and Theriogenology, Faculty of Veterinary, Animal and Biomedical Sciences, Sylhet Agricultural University, Sylhet, Bangladesh; 8 Department of Clinical Sciences, Swedish University of Agricultural Sciences, Uppsala, Sweden; 9 Swedish Veterinary Agency, Uppsala, Sweden; Beni Suef University Faculty of Veterinary Medicine, EGYPT

## Abstract

A cross-sectional study was conducted to estimate the prevalence of intramammary infection (IMI) associated bacteria and to identify risk factors for pathogen group-specific IMI in water buffalo in Bangladesh. A California Mastitis Test (CMT) and bacteriological cultures were performed on 1,374 quarter milk samples collected from 763 water buffalo from 244 buffalo farms in nine districts in Bangladesh. Quarter, buffalo, and farm-related data were obtained through questionnaires and visual observations. A total of 618 quarter samples were found to be culture positive. Non-*aureus* staphylococci were the predominant IMI-associated bacterial species, and *Staphylococcus* (*S*.) *chromogenes*, *S*. *hyicus*, and *S*. *epidermidis* were the most common bacteria found. The proportion of non-aureus staphylococci or *Mammaliicoccus sciuri* (NASM), *S*. *aureus*, and other bacterial species identified in the buffalo quarter samples varied between buffalo farms. Therefore, different management practices, buffalo breeding factors, and nutrition were considered and further analyzed when estimating the IMI odds ratio (OR). The odds of IMI by any pathogen (OR: 1.8) or by NASM (OR: 2.2) was high in buffalo herds with poor milking hygiene. Poor cleanliness of the hind quarters had a high odds of IMI caused by any pathogen (OR: 2.0) or NASM (OR: 1.9). Twice daily milking (OR: 3.1) and farms with buffalo purchased from another herd (OR: 2.0) were associated with IMI by any pathogen. Asymmetrical udders were associated with IMI-caused by any bacteria (OR: 1.7). A poor body condition score showed higher odds of IMI by any pathogen (OR: 1.4) or by NASM (OR: 1.7). This study shows that the prevalence of IMI in water buffalo was high and varied between farms. In accordance with the literature, our data highlight that IMI can be partly controlled through better farm management, primarily by improving hygiene, milking management, breeding, and nutrition.

## Introduction

Subclinical mastitis (SCM) is the inflammation of the mammary gland mainly in response to intra-mammary infection (IMI) in water buffalo [1–3]. Subclinical mastitis significantly affects food security and safety, as it reduces milk yield and negatively affects the quality of milk and milk products [4, 5], increasing the culling rate and the risk of the development and spread of antimicrobial resistance [6, 7]. Knowing the risk factors for IMI is essential for determining the most appropriate control measures for IMI in water buffalo.

Previous studies have attempted to identify several buffalo and farm level risk factors for buffalo SCM based on positive reaction in California mastitis test (CMT). Animal-related risk factors reports include age, breed, parity, and lactation stage in South Asian countries, e.g., Pakistan and India [8–10]. Among individual water buffalo, a distinct morphological variation usually exists in udder and teat shapes. The udder may be bowl-shaped, globular, or pendulous, whereas the teat may be cylindrical, conical, or funnel-shaped [11, 12]. These variations in udder morphology may also influence the entry and establishment of IMI into the mammary gland. In household buffalo farms, hand milking is a common milking practice in most South Asian countries. Therefore, trauma caused by incorrect milking procedures may also be responsible for IMI in these animals [13]. Examples of farm-related risk factors for SCM for water buffalo are bedding materials, the rearing system used, and the type of milking system [9, 14]. Poor hygiene and management usually contribute to the exposure of the mammary gland to pathogens, resulting in a high prevalence of IMI. Although several risk factors for positive CMT have been identified, few studies have investigated the risk factors for different pathogens associated with IMI in water buffalo.

Previous studies reported that non-aureus Staphylococci are frequently isolated in milk samples from buffalo quarters, followed by *Staphylococcus* (*S*.) *aureus* and *Streptococcus* spp, and are also associated with several risks, such as virulence factors and developing antimicrobial resistance [3, 15, 16]. Intramammary infection is caused by a wide variety of bacteria species, and species-level differential is challenging using bacteriological culture and classical phenotypical tests; for example, species of non-aureus Staphylococci are often misidentified [17, 18]. Previous studies reported that bacterial identification is possible using MALDI-TOF MS with better accuracy [18]. But to date, knowledge regarding species-level identification of buffalo IMI-causing bacteria is minimal.

Identifying aetiological agents and associated risk factors for IMI is important for developing effective management practices to control buffalo SCM in Bangladesh; these practices may also be applicable in buffalo-concentrated neighboring Asian countries with similar management, e.g., India, Pakistan, Iran, Nepal, and Indonesia [19–22]. To represent the udder health and milk quality in water buffalo, two previous studies have been published in this project describing the prevalence and risk factors of SCM and bulk milk somatic cell count in Bangladesh [12, 23]. Hence, this study aimed to estimate the prevalence of IMI-associated bacterial species and identify risk factors for pathogen group-specific IMI in water buffalo in Bangladesh.

## Materials and methods

### Ethics statement

The study was approved and performed in line with guidelines by the Animal Experimentation Ethics Committee of the Sylhet Agricultural University research system (Ethical approval number: #AUP2021006). The farmers gave written informed consent during the interview. Milk samples were collected: being a non-invasive sampling process and requiring no anesthesia or euthanasia, further approval was not required.

## Study area description

This cross-sectional study was conducted in nine representative buffalo-concentrated districts in Bangladesh between February 2020 and April 2021. The geographical location of the selected districts are as follows: Noakhali and Chattogram in the south-east, Lakshmipur and Bhola in the south-central region, Maulvibazar and Mymensingh in the north-east, Jamalpur in the north-central region, Rajshahi district in the north-west, and Dhaka, located in the center of Bangladesh. The geographical locations have been displayed on a map in a previously published study of this project [12]. Buffalo-rearing systems in these regions can be subdivided into five different buffalo-rearing systems: free-ranging, semi-free-ranging, intensive, semi-intensive, and household. Free-range buffalo graze on the fallow land in coastal or semi-coastal islands, supplemented with straw or small amounts of concentrates. Buffalo are transported across islands in a free-ranging system depending on green roughage availability. Due to the feed scarcity on the islands during the dry season (October to March), buffalo are relocated inland for 3–6 months in the semi-free-range system, depending on roughage availability. The household rearing system allows 5–7 hours of grazing, supplemented with a small amount of straw, grass, and concentrates. In river-basin locations, the semi-intensive system involves keeping buffalo in sheds, grazing them on nearby pastures, and returning them to the sheds each day. Buffalo in intensive systems are tethered in sheds. They are stall-fed and never permitted to graze. In this rearing system, buffalo are fed with cultivated fodder and a concentrate mixture prepared on the farm.

### On-farm data collection

A questionnaire was developed, and the collected data on the farm and animal level information used in the previous two studies to assess the prevalence and risk factors of SCM and bulk milk somatic cell count [3, 23] was also used in this study. In these two previous studies, risk factor data was similar but the outcome variable was different than this study. The data was collected through a face-to-face interview with the farmer, and on-farm observations were taken. During the interview, the farmers gave buffalo farm information on the buffalo source (stock, stock or purchased, purchased or contract), breed type (indigenous or cross breed), buffalo type (river type, swamp type or mixed), and frequency of milking per day (once or twice). In contrast, animal information, such as body condition score in the scale 1 to 9 (poor: score 1 to 3, moderate: score 4, good: score 5, and fat: score 6 to 9), udder symmetry (symmetrical or asymmetrical), udder shape (cup or pendulous, round or globular, or bowl), type of hand milking procedure (using full hand, stripping, or knuckling), cleanliness of the hind quarter (excellent, good or poor), and milking hygiene (excellent, good, or fair), was assessed through on-farm observations by the interviewer. The questionnaire used has been detailed in a previously published study [12].

### Detection of subclinical mastitis using a California mastitis test and collection of quarter milk samples

A California Mastitis Test (CMT) was performed on the individual buffalo udder quarters with no visible signs of clinical mastitis. The test results were scored from 1 to 5 and classified as being either healthy (CMT score 1) or being a SCM quarter (CMT scores 2 to 5) [12, 24]. Milk samples from all the healthy and SCM quarters were taken following aseptic milk sampling procedures [24] in 15 mL sterile Falcon tubes. Milk samples were temporarily stored in a freezer (-10°C to -15°C) near the field visit area. Within a week, they were transferred to the Udder Health Bangladesh bacteriological laboratory of Chattogram Veterinary and Animal Sciences University and stored at -20°C.

### Bacteriological culture

Milk samples were subjected to bacteriological culture in the Udder Health Bangladesh laboratory following NMC guidelines [24], with some modifications noted below [25]. Quarter milk samples were inoculated (10 μL) using 5% bovine blood agar. Positive growth was defined when at least three morphologically similar colonies were present on blood agar. At the same time, a sample was considered to be contaminated when it yielded three or more morphologically different colony types on blood agar. Gram-positive and Gram-negative bacteria were differentiated based on growth characteristics, followed by inoculation using selective agar media: Mannitol Salt agar, MacConkey agar, and Eosin Methylene Blue agar. Bacterial isolates were preserved at -80°C using a brain heart infusion broth and 50% buffered glycerol for future use. All bacterial culture media and reagents were manufactured by Oxoid (Oxoid Ltd, Basingstoke, United Kingdom).

#### Matrix-assisted laser desorption/ionization time-of-flight mass spectrometry

Pure bacterial cultures were preserved adding 700 μL culture to 300 μL 50% buffered glycerol in 1.5 mL sterile Cryogenic Vials (Thermo Fisher Scientific Inc, Massachusetts, USA) shipped (Italian Ministry of Health Authorization number: 477015235) to the Animal Infectious Diseases Laboratory, Department of Veterinary and Animal Sciences at the Università degli Studi di Milano for further confirmation, using MALDI-TOF MS. For MALDITOF-MS identification, all frozen bacterial isolates were inoculated (10 μL) using blood agar and incubated for 24 hours. The pure cultures were further sub-cultured to ensure the complete bacterial function of the isolates. One or two colonies, based on very small or large colony size, e.g., one colony for *Staphylococcus* spp. and two colonies for *Streptococcus* spp., were deposited on the target plate using a toothpick overlaid with 1 μL of α-cyano-4-hydroxycinnamic acid (Bruker Daltonics, GmbH, Bremen, Germany) and were dried. The spectra were acquired using a microFlex™ mass spectrometer (Bruker Daltonics, GmbH). The results were interpreted by comparing them against the MBT Compass® 4.1 database [26]. Log scores of ≥ 2.0 were the thresholds used for species confirmation of *S*. *aureus*. In contrast, log scores of ≥ 1.7 were used to confirm the species identification of non-aureus staphylococci or *Mammaliicoccus* spp. (NASM) [17, 18]. If a pure culture was not identifiable in MALDI-TOF using a second subculture, it was defined as “Not identifiable”. “Other bacterial species” was defined if a pure culture was identified in MALDI-TOF as Not identifiable or Gram-negative bacteria species such as, *Pseudomonas* spp. *Acinetobacter* spp. *Enterobacter* spp., *Escherichia coli*, *Stenotrophomonas* spp., *Klebsiella* spp. *Ochrobactrum* spp. or *Micrococcus* spp., *Corynebacterium* spp. or *Rothia* spp. or *Lactococcus garvieae* or *Bacillus* spp. or, *Kocuria* spp. or, *Actinomyces* spp. or *Arcanobacterium* spp. or *Microbacterium* spp. or *Rhodococcus* spp. or *Brachybacterium* spp., and *Streptococcus* spp.

### Bulk milk somatic cell count

Bulk milk was collected on the same farms on the same day as the test samples. SCC was measured in thoroughly mixed morning bulk milk from all the lactating buffalo at each farm using a DeLaval somatic cell counter (DCC) (DeLaval Group, Stockholm, Sweden) [24]. The procedure was carried out according to the manufacturer’s instructions, described in a previous study from this project [3].

### Definition of subclinical mastitis at the quarter, buffalo, and farm level

A buffalo was considered SCM-positive when one or more functional quarters had CMT ≥ 2. A farm was considered SCM positive when at least one buffalo had a quarter with CMT ≥ 2. The definition was set following a previous study from this project [12].

### Statistical analysis

Data analysis was done using R statistical software (version 4.1.2; R Foundation for Statistical Computing, Vienna, Austria). The prevalence of IMI in healthy (CMT = 1) or SCM quarter (CMT ≥ 2) samples was calculated by dividing the number of samples positive for each bacterial species (NASM; *S*. *aureus*; *Streptococcus*; Gram-negative; or other bacteria) by the total number of tested quarter milk samples. The exact binomial method was used to calculate the proportion’s 95% confidence intervals. The prevalence of IMI-associated bacterial species was calculated at the quarter, animal, and farm levels. Mixed-effects logistic regression models with animal and farm ID as random effects were constructed to test the association between IMI-associated bacterial species and buffalo or farm-level variables. The significance of the random effect terms was checked using a Likelihood Ratio Test using the latent variable approach [27]. Variables for which the univariable *P* value was < 0.20 were included in the multivariable model. The multivariable model was built using a stepwise forward selection procedure, adding each significant variable in the model, starting with the variable with the lowest *P* value. If the beta coefficient in the new model changed by more than 30% after adding a variable, this was deemed confounding, and the confounder was included in the model. If the standard error in the new model largely changed, it indicated collinearity with the newly added variable, and the most biologically meaningful variable for buffalo IMI was kept in the final model. Finally, the association of significant (*P* ≤ 0.05) variables with the outcome was presented in terms of subject-specific odds ratios (OR) and 95% CI, meaning that the fixed effects in a mixed model represented the effects of that factor within the cluster [27].

## Results

### Descriptive statistics

The numbers of lactating animals in the herds varied between 1 and 46 (Mean 7.4; Standard deviation 6.9). Table 1 shows that 1,374 quarters from the 763 lactating buffalo from 244 buffalo farms from nine districts were included in the analysis. The prevalence of quarters with CMT = 1 was 49% (95% CI, 46–52), and CMT ≥ 2 quarters was 51% (95% CI, 48–54). The prevalence for each district is displayed in Table 1.

**Table 1:** Distribution of quarter milk samples collected and analyzed for bacteriological culture in nine water buffalo-concentrated districts in Bangladesh.

Districts	N farms	N buffalo	N quarters	CMT = 1		CMT ≥ 2	
				N quarters	% (95% CI)	N quarters	% (95% CI)
Noakhali	45	168	292	145	49.7 (43.8 to 55.5)	147	50.3 (44.5 to 56.2)
Jamalpur	35	134	224	117	52.2 (45.5 to 58.9)	107	47.8 (41.1 to 54.5)
Rajshahi	39	104	191	89	46.6 (39.4 to 53.9)	102	53.4 (46.1 to 60.6)
Bhola	37	86	186	89	47.8 (40.5 to 55.3)	97	52.2 (44.7 to 59.5)
Chattogram	38	93	160	92	57.5 (49.4 to 65.3)	68	42.5 (34.7 to 50.6)
Moulvibazar	37	94	146	82	56.2 (47.7 to 64.4)	64	43.8 (35.6 to 52.3)
Laxmipur	2	47	103	34	33.0 (24.1 to 43.0)	69	67.0 (57.0 to 75.9)
Mymensingh	10	23	45	16	35.6 (21.9 to 51.2)	29	64.4 (48.8 to 78.1)
Dhaka	1	14	27	10	37.0 (19.4 to 57.6)	17	63.0 (42.4 to 80.6)
**Total**	**244**	**763**	**1,374**	**674**	49.1 **(46.4 to 51.7)**	**700**	50.9 (**48.3 to 53.6)**

### Prevalence of IMI-associated pathogens

Table 2 shows the quarter, buffalo, and farm-level prevalence of non-aureus staphylococci, *Mammaliicoccus* spp., other bacterial species, and *S*. *aureus*. Non-aureus staphylococci or *Mammaliicoccus* spp. were found to be the most prevalent bacterial species at the quarter (32%), followed by animal (45%) and farm levels (72%).

**Table 2:** Herd, buffalo, and quarter-level prevalence of intramammary infection in 1,374 buffalo quarters from 763 buffalo from 244 water buffalo farms in nine buffalo-concentrated districts in Bangladesh.

Culture positive regardless of CMT status	N quarters (%)^a^	N buffalo (%)^b^	N farms (%)^c^
Non-aureus staphylococci or *Mammaliicoccus* spp.	439 (32.0)	341 (44.5)	176 (72.1)
Other bacterial species^d^	140 (10.2)	129 (16.9)	91 (37.3)
*Staphylococcus aureus*	39 (2.8)	37 (4.9)	32 (13.1)
Total	618 (45.0)	462 (60.6)	209 (85.7)

^a^ Contaminated samples (n = 11) were excluded from the statistical analysis.

^b^ A buffalo was considered positive when at least one of the quarters had an IMI.

^c^ A buffalo farm was considered positive when at least one buffalo had an IMI.

^d^ Other bacterial species included non-identifiable bacteria species, Gram-negative bacteria species, *Micrococcus* spp., *Corynebacterium* spp., *Rothia* spp., *Lactococcus garvieae*, *Bacillus* spp., *Kocuria* spp., *Actinomyces* spp., *Arcanobacterium* spp., *Microbacterium* spp., *Rhodococcus* spp., *Brachybacterium* spp., and *Streptococcus* spp.

**Fig 1:**
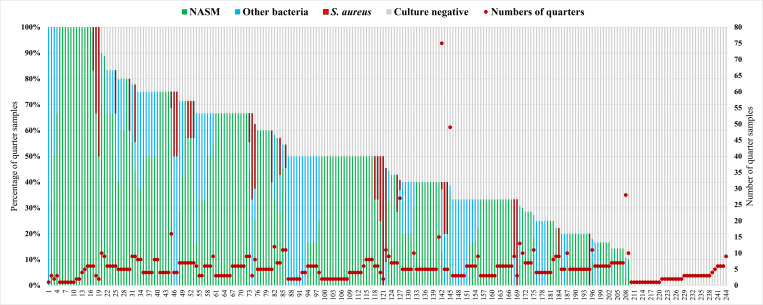
Distribution of culture results for intramammary infection causing pathogen per farm. Distribution of culture results for 1,374 buffalo quarter milk samples from 763 buffalo from 244 water buffalo farms in nine concentrated buffalo districts in Bangladesh, sorted by the percentage of culture-negative samples, along with the total number of samples per farm. NASM stands for non-aureus staphylococci or *Mammaliicoccus* spp.

On average, six-quarters of the samples were examined for bacteriological culture per farm (median: 5; range: 1–75). Fig 1. shows how the distribution of IMI-associated pathogens per farm and the proportion of culture negatives varied widely across the buffalo farms.

### Prevalence of IMI-associated pathogens

In total, 618 quarters were culture-positive, 756 were culture-negative, and 11 samples were found to be contaminated. When considering the buffalo quarters with CMT = 1, 46% (312/674) (95% CI, 43–50) were culture-positive; when CMT was ≥ 2, bacteria were isolated from 44% (306/700) (95% CI, 40–48) of the quarters. Bacterial isolates belonging to the *Staphylococcus* genus were common, where *S*. *chromogenes* was the most prevalent species, followed by *S*. *hyicus*, *S*. *aureus*, and *S*. *xylosus*. The pathogen distribution is given in Table 3.

**Table 3:** Distribution of bacterial species from intramammary infections (N = 618) isolated from 1,374 quarter milk samples from 763 lactating water buffalo from 244 buffalo farms in nine water buffalo-concentrated districts in Bangladesh. Percentages were calculated within CMT classes.

Bacterial species	CMT = 1 quarters n (%)	CMT ≥ 2 quarters n (%)	Total quarters^a^ n (%)
**Staphylococcus**			
*S*. *chromogenes*	60 (8.9)	66 (9.4)	126 (9.17)
*S*. *hyicus*	53 (7.9)	60 (8.6)	113 (8.22)
*S*. *aureus*	19 (2.8)	20 (2.9)	39 (2.83)
*S*. *xylosus*	18 (2.7)	12 (1.7)	30 (2.18)
*S*. *epidermidis*	21 (3.1)	9 (1.3)	30 (2.18)
*S*. *haemolyticus*	15 (2.2)	14 (2.0)	29 (2.11)
*S*. *arlettae*	6 (0.9)	1 (0.1)	7 (0.50)
*S*. *pasteuri*	2 (0.3)	4 (0.6)	6 (0.43)
*S*. *hominis*	1 (0.1)	2 (0.3)	3 (0.21)
*S*. *equorum*	1 (0.1)	1 (0.1)	2 (0.14)
*S*. *warneri*	1 (0.1)	1 (0.1)	2 (0.14)
*S*. *ureilyticus*	2 (0.3)	-	2 (0.14)
*S*. *saprophyticus*	1 (0.1)	-	1 (0.07)
*S*. *casei*	1 (0.1)	-	1 (0.07)
*S*. *capitis*	-	1 (0.1)	1 (0.07)
*S*. *kloosii*	-	1 (0.1)	1 (0.07)
***Mammaliicoccus***			
*Mammaliicoccus sciuri*	39 (5.8)	46 (6.6)	85 (6.18)
**Streptococcus**			
*Strep*. *uberis*	2 (0.3)	1 (0.1)	3 (0.21)
*Strep*. *suis*	1 (0.1)	1 (0.1)	2 (0.14)
*Strep*. *canis*	1 (0.1)	1 (0.1)	2 (0.14)
*Strep*. *dysgalactiae*	1 (0.1)	1 (0.1)	2 (0.14)
*Strep*. *pluranimalium*	-	1 (0.1)	1 (0.07)
**Gram-negative**			
*Pseudomonas* spp.	1 (0.1)	4 (0.6)	5 (0.36)
*Acinetobacter* spp.	1 (0.1)	1 (0.1)	2 (0.14)
*Enterobacter* spp.	1 (0.1)	1 (0.1)	2 (0.14)
*Escherichia coli*	1 (0.1)	1 (0.1)	2 (0.14)
*Stenotrophomonas* spp.	-	2 (0.3)	2 (0.14)
*Klebsiella* spp.	-	1 (0.1)	1 (0.07)
*Ochrobactrum* spp.	-	1 (0.1)	1 (0.07)
**Other bacteria**			
*Micrococcus* spp.	13 (1.9)	11 (1.6)	24 (1.74)
*Corynebacterium* spp.	8 (1.2)	7 (1.0)	15 (1.09)
*Rothia* spp.	5 (0.7)	5 (0.7)	10 (0.72)
*Lactococcus garvieae*	2 (0.3)	3 (0.4)	5 (0.36)
*Bacillus* spp.	4 (0.6)	1 (0.1)	5 (0.36)
*Kocuria* spp.	2 (0.3)	2 (0.3)	4 (0.29)
*Actinomyces* spp.	2 (0.3)	1 (0.1)	3 (0.21)
*Arcanobacterium* spp.	-	2 (0.3)	2 (0.14)
*Microbacterium* spp.	1 (0.1)	-	1 (0.07)
*Rhodococcus* spp.	1 (0.1)	-	1 (0.07)
*Brachybacterium* spp.	-	1 (0.1)	1 (0.07)
Not identifiable^b^	25 (3.7)	19 (2.7)	44 (3.20)
Culture negative	362 (53.7)	394 (56.3)	756 (55.0)
**Total quarters examined**	**674 (100)**	**700 (100)**	**1374 (100)**

^a^ Contaminated samples (n = 11) were excluded from the statistical analysis.

^b^ Not identifiable: Bacterial species could not be confirmed through MALDI-TOF MS with log scores of ≥ 1.7.

### Risk factors for intramammary infection

Four variables, such as type of buffalo breed, udder shape, type of hand milking, and bulk milk somatic cell count, were non-significant in the univariable analysis. In this study, the buffalo source was related to the type of buffalo. Farms with stock buffalo frequently had river-type buffalo instead of swamp or mixed (river and swamp type). In this study, the river-type buffalo had higher odds of IMI by non-aureus Staphylococci than the swamp-type buffalo. Animal-related risk factors for water buffalo IMI were the cleanliness of the hind quarter, body condition score, and udder symmetry. Buffalo farm-related risk factors were milking hygiene, frequency of milking per day, and buffalo source. Poor milking hygiene was associated with the risk of IMI by any bacterial species and NASM. A poor cleanliness score for hind quarters was found to be a risk factor for IMI by any bacterial species and NASM. Asymmetrical udders were associated with IMI by any bacterial species. Buffalo born on the farm (own-stock) had, on average, lower body condition scores than purchased buffalo, meaning body condition scores confounded the association of buffalo source with IMI by any pathogens. The district was associated with the buffalo-rearing system. We kept the rearing system in the model because this is biologically easier to interpret than the effects of districts in Bangladesh. However, the rearing system was statistically non-significant and was excluded from the final model in the model-building process. In the model of IMI caused by NASM, bulk milk somatic cell count and the type of hand milking system were collinear with milking hygiene; thus, milking hygiene was chosen to remain in the final model. Table 4 shows the factors associated with IMI caused by any bacterial species and NASM.

**Table 4:** Mixed effect multivariable logistic regression analysis using two separate models for intramammary infection (IMI) by any pathogen, non-aureus staphylococci or *Mammaliicoccus sciuri* regressed against buffalo and farm-level risk factors, using animal and farm as the random effects, based on bacteriological culture data from 1,374 quarters from 763 buffalo in 244 farms in nine buffalo-concentrated districts in Bangladesh. Subject-specific odds ratios and 95% CI were calculated based on subject-specific beta estimates.

	Any bacterial species^a^		NASM^b^	
**Independent variable**	**Odds ratio**	**95% CI**	**Odds ratio**	**95% CI**
**Milking hygiene**				
Excellent	Reference			
Poor	1.8	1.3 to 2.5	2.2	1.5 to 3.2
**Cleanliness of the hind quarters**				
Excellent	Reference			
Good	1.5	1.1 to 2.0	1.4	1.0 to 1.9
Poor	2.0	1.4 to 2.7	1.9	1.3 to 2.8
**Frequency of milking per day**				
Once	Reference			
Twice	3.1	2.0 to 4.6	-	-
**Buffalo source**				
Purchased from other herds	Reference			
Source unknown	1.3	0.4 to 3.9	-	-
Both own stock or purchased	1.5	0.9 to 2.3	-	-
Own stock	2.0	1.2 to 3.2	-	-
**Udder symmetry**				
Symmetrical	Reference			
Asymmetrical	1.70	1.0 to 2.8	-	-
**Body condition score (1–9)**				
Good (5)	Reference			
Fatty (6–9)	1.1	0.8 to 1.5	1.0	0.6 to 1.7
Moderate (4)	1.1	0.8 to 1.5	1.4	1.0 to 2.0
Poor (1–3)	1.4	1.0 to 1.9	1.7	1.1 to 2.5

^a^ IMI by any bacterial species: The subject-specific odds ratios were calculated from 1,233 quarters from 681 lactating buffalo from 217 farms.

^b^ IMI by non-aureus staphylococci or *Mammaliicoccus sciuri*: The subject-specific odds ratios were calculated from 1,287 quarters from 727 lactating buffalo from 229 farms.

## Discussion

This study aimed to determine the prevalence of IMI and identify risk factors for IMI-associated bacterial species in water buffalo in Bangladesh. This study demonstrated the predominance of NASM (32%) in IMI in water buffalo quarters in Bangladesh, which is in agreement with our previous work [3], where we found a prevalence of 25% (95% CI 18–33) in water buffalo in Bangladesh. Several previous studies in India also demonstrated non-aureus Staphylococci as the most prevalent among the culture results (18–35%) in SCM quarters in water buffalo [10, 28, 29]. *Staphylococcus chromogenes* were the most common among NASM species in the present study, followed by *S*. *hyicus* and *Mammaliicoccus sciuri*. This species was formerly known as *S*. *sciuri* but has recently been reclassified as “*Mammaliicoccus sciuri*” [18, 30] and has been previously reported in water buffalo [7, 31].

Non-aureus staphylococci may colonize the teat canal of the buffalo mammary gland [32, 33] but are also known to be associated with SCM and occasionally with clinical mastitis in various dairy species, such as cows, buffalo, and goats [34–37]. Surprisingly, our study found a similar prevalence of positive culture results in CMT-positive and healthy quarters, consistent with our previous study [3]. If the culture of NASM results from true IMI rather than teat canal colonization in water buffalo, the prevalence of non-aureus Staphylococci would be expected to be higher in SCM quarters than in healthy quarters, as previously reported in water buffalo and dairy cows [38–40]. Our data suggests that part of the NASM we cultured may have originated from teat canal colonization rather than IMI. The absence of a difference in the proportion of culture positives between healthy and CMT-positive quarters may also be explained through the interdependence of microbiota among the quarters of an individual buffalo [41]. It has also been shown in dairy cows that an IMI in one single quarter may also evoke an inflammatory response in the other quarters of that animal [42, 43]. To what extent the unaffected quarters react may depend on the pathogenicity of the pathogen and the severity of the inflammation [44–46]. Therefore, the homolateral quarter of bacteriologically positive CMT-positive quarters might be CMT-positive and bacteriologically negative because of an inflammatory response in the other quarter.

The prevalence of IMI-causing bacterial species in the buffalo quarters largely varied between farms (Fig 1). In a previous study on dairy cows, within farms, the cow-level prevalence of IMI by NASM ranged from 0 to 50% [47], and the prevalence of *S*. *aureus* varied from 0 to 73% [48]; for water buffalo, however, no previous work is available to our knowledge. In an earlier study, we found a significant variation in the prevalence of SCM at the animal and quarter levels between the buffalo farms, which is also evidenced in our study’s IMI. However, herd level variation needs to be cautiously interpreted. In fact, prevalence between herds is not entirely comparable because of a large variation in number of lactating buffalo in a herd, as in a 3-animal herd, 1 positive means 33% prevalence. Therefore, there is a bit more uncertainty in the smaller herds. These differences between farms may result from different management or the virulence of the dominant infecting strains in a herd [48–50]. Genotyping of IMI pathogens may help elucidate the potential of various strains and their transmission routes. A previous study using a bioeconomic simulation model demonstrated how the characterization of *S*. *aureus* strains could be advantageous for identifying the outcome of most intervention strategies [51]. Therefore, to understand the mechanism of IMI-associated pathogen transmission in water buffalo, genotyping of the most important species may be a valuable next step.

Buffalo with poor hind quarter cleanliness had higher odds of IMI by any bacterial species and NASM than buffalo with clean hind quarters. Sub-optimal hygienic conditions in the udder and hind quarter may expose the teat canal to pathogens and result in an IMI [52]. Contamination of hind quarters predisposes the buffalo to environmental IMI pathogens [53, 54]. Since dirt is unlikely to be the primary source of NASM-associated IMI, we speculate that dirty hindquarters may be linked to other factors predisposing the animal to an IMI, such as the rearing system or general hygiene. In this study, the hind quarters were dirtier in semi-free-ranging buffalo farms and buffalo with poor milking hygiene. This suggests that washing buffalo will not effectively reduce the prevalence of NASM in water buffalo in Bangladesh. Instead, the underlying risk factors should be identified in future studies.

Poor milking hygiene, such as farmers not washing their hands and not using antiseptics or teat dipping before or after milking, was associated with higher odds of IMI by any bacterial species than excellent milking hygiene. This finding is consistent with a study in Brazil, which evidenced that poor milking hygiene was associated with IMI and SCC > 400,000 cells per mL of milk in water buffalo quarters [55]. In a review article, poor floor hygiene, inaccurate hand milking procedures, and dirty milkers’ hands were reported to be associated with SCM or IMI in dairy cows in South Asian countries [56]. In our study, farms with poor milking hygiene frequently milked using stripping. This hand milking method involves the knuckling method, which consists of bending the thumb and pressing the teat against the index finger with the nail and end of the thumb, which may cause injury to the teat skin and inside the teat canal. The teat skin and sphincter serve as the first line of defense to protect against mastitis pathogens. When the teat is injured, it may lose this protective function, making it susceptible to infection [57, 58]. Thus, milking technique and hygiene should be addressed in udder health control efforts.

In this study, buffalo with an asymmetrical udder had higher odds of IMI by any pathogen than buffalo with symmetrical udders. *Staphylococcus aureus* is known to cause chronic subclinical inflammation in the water buffalo mammary gland [59], which may result in udder fibrosis and shrinkage of the teat cistern [60]. In our study, a significantly higher proportion of buffalo with an asymmetrical udder had a history of clinical mastitis than those with symmetrical udders, suggesting clinical mastitis may also result in udder gland atrophy.

Surprisingly, in this study, own-stock buffalo farms had higher odds of IMI by any pathogen than farms with purchased buffalo. The source of buffalo may be linked with other factors predisposing the animals to an IMI. River-type buffalo generally have a high milk yield. The river-type buffalo had a higher odds of IMI by non-aureus Staphylococci than the swamp-type buffalo, suggesting that milk yield was possibly underlying this association. A previous study on dairy cows reported that teat canal diameter and stretchability are correlated with milk production level [61]. River-type buffalo generally produce more milk, and the teat canal may remain open longer, as evidenced in high-yielding dairy cows [62]; thus, this may increase the risk of IMI pathogens entering the teat.

## Conclusions

In conclusion, several udder health problems related to IMI in water buffalo have been identified in this study. NASM was the most common species group responsible for IMI in water buffalo in Bangladesh. We have shown that the prevalence of IMI-associated bacteria varies between farms. Strain typing of the IMI-associated bacterial species may help elucidate the transmission routes of the major pathogens. Our data suggests that IMI can be partly addressed through better farm management, including improved milking techniques and better hygiene, which can be used to design water buffalo udder health control strategies.

## Supporting information

S1 File (DOCX)

S2 File (DOCX)
