# Correction: Pathogen group-specific risk factors for intramammary infection in water buffalo

**DOI:** 10.1371/journal.pone.0334533

**Published:** 2025-10-13

**Authors:** Shuvo Singha, Gerrit Koop, Md. Mizanur Rahman, Fabrizio Ceciliani, Maria Filippa Addis, Md. Matiar Rahman Howlader, Mohammed Kawser Hossain, Renata Piccinini, Clara Locatelli, Ylva Persson, Valerio Bronzo

In the Abstract, there are errors in the tenth sentence. The correct sentence is: Twice daily milking (OR: 3.1) and own stock buffalo (OR: 2.0) were associated with IMI by any pathogen.

In the Funding Statement, there is an error in Dr. Ylva Persson’s listed affiliation. The correct affiliation is: Dr. Ylva Persson is currently working as State Veterinarian in Swedish Veterinary Agency is the author who was awarded the grant.
