# Supplementary material for: Pathogen group-specific risk factors for intramammary infection in water buffalo
Source: PLoS One. 2024 Apr 4;19(4):e0299929. doi: 10.1371/journal.pone.0299929 (PMC10994383; doi:10.1371/journal.pone.0299929)
Supplement: S1 File — (DOCX) [file pone.0299929.s001.docx]

**Response to the JOURNAL REQUIREMENTS:**

1. Please ensure that the author list and affiliations are correct on the title page of your manuscript, and that your author contributions, competing interests, and financial disclosure are correct as listed below. All of these sections will be indexed in PubMed and published by PLOS ONE as you have written them. Please email [plosone@plos.org](mailto:plosone@plos.org) if any changes to this content need to be made.

Please see here for the full list and definition of contributor roles: [https://eur01.safelinks.protection.outlook.com/?url=http%3A%2F%2Fjournals.plos.org%2Fplosone%2Fs%2Fauthorship%23loc-author-contributions&data=05%7C02%7Cylva.persson%40sva.se%7C794a351996ed4856452a08dc328b1d42%7Cb64756aa7df04f2ea41171214a50af24%7C0%7C1%7C638440822087742055%7CUnknown%7CTWFpbGZsb3d8eyJWIjoiMC4wLjAwMDAiLCJQIjoiV2luMzIiLCJBTiI6Ik1haWwiLCJXVCI6Mn0%3D%7C0%7C%7C%7C&sdata=7CoNvH5wb7LInhR6pIVKih8v9wG4o7SLkftHxjfAlT4%3D&reserved=0](http://journals.plos.org/plosone/s/authorship#loc-author-contributions)

**Response**

Yes, the authors are affiliated correctly and their contributions are mentioned accurately.

3. Please ensure that the Competing Interests and Financial Disclosure statements listed below are suitable for publication. These sections will be indexed in PubMed and published by PLOS ONE as you have written them. Please email [plosone@plos.org](mailto:plosone@plos.org) if any changes to these statements need to be made.

Competing Interests:
The authors have declared that no competing interests exist

Financial Disclosure:
Dr. Ylva Persson is currently working as State Veterinarian in National Veterinary Institute Sweden is the author who was awarded the grant.

The grant was awarded by Swedish Research Council The Vetenskapsrådet (grant number: 2018–03583)

**Response**

This information is correct and agreed by the authors

4. Funder website: [https://eur01.safelinks.protection.outlook.com/?url=https%3A%2F%2Fwww.vr.se%2Fenglish&data=05%7C02%7Cylva.persson%40sva.se%7C794a351996ed4856452a08dc328b1d42%7Cb64756aa7df04f2ea41171214a50af24%7C0%7C1%7C638440822087747988%7CUnknown%7CTWFpbGZsb3d8eyJWIjoiMC4wLjAwMDAiLCJQIjoiV2luMzIiLCJBTiI6Ik1haWwiLCJXVCI6Mn0%3D%7C0%7C%7C%7C&sdata=%2BGluxC9uWHWrltmgmRVnzzYbRD20WNLfYnlolOMbc0Q%3D&reserved=0](https://www.vr.se/english)

No, the funder did not play any role in the study design, data collection and analysis, decision to publish, or preparation of the manuscript.

**Response**

This information is correct and agreed by the authors

5. To prevent production delays, we recommend using the Author Formatting Checklist to confirm that your paper meets PLOS ONE's typesetting requirements for References, Tables, and Figures: [https://eur01.safelinks.protection.outlook.com/?url=http%3A%2F%2Fjournals.plos.org%2Fplosone%2Fs%2Ffile%3Fid%3Dc819%2Fplos-one-author-formatting-checklist.docx&data=05%7C02%7Cylva.persson%40sva.se%7C794a351996ed4856452a08dc328b1d42%7Cb64756aa7df04f2ea41171214a50af24%7C0%7C1%7C638440822087752612%7CUnknown%7CTWFpbGZsb3d8eyJWIjoiMC4wLjAwMDAiLCJQIjoiV2luMzIiLCJBTiI6Ik1haWwiLCJXVCI6Mn0%3D%7C0%7C%7C%7C&sdata=C1ZE0jNw3USrDEnT7XZr%2FjIZ0tK31vooPZ9Oj9iIi4I%3D&reserved=0](http://journals.plos.org/plosone/s/file?id=c819/plos-one-author-formatting-checklist.docx).

This checklist is a reference tool for you; please do not upload the completed Author Formatting Checklist with your submission files.

**Response**

[**References**](https://journals.plos.org/plosone/s/submission-guidelines#loc-references)

Is every item in the reference list cited in the main text? The reference list should only contain items cited in the main text. (References cited only in the Supporting Information should appear in a separate reference list within the supporting information.)

Are all reference citations denoted using the reference number in square brackets? Please do not use an author-year citation style.

Do all reference citations appear in the manuscript in ascending numerical order?

[**Tables**](https://journals.plos.org/plosone/s/tables)

Are all tables editable, cell-based objects? Tables in graphic format cannot be typeset. (Any graphic items must be submitted as figures or as supporting information.)

Are all tables cited in numeric order within the main text?

[**Figures**](https://journals.plos.org/plosone/s/figures)

Are your figures clear and legible, including the text? Do they follow PLOS figure requirements?

Does each figure match its corresponding caption and citation in the main text?

Are all figures cited in numeric order?

[**Supporting Information**](https://journals.plos.org/plosone/s/supporting-information)

Are your Supporting Information files clear of tracked changes? Supporting Information files will be published exactly as provided.

If your paper includes Appendices, are they included as Supporting Information files? Appendices must be within the SI and not the main manuscript text.

For more information about formatting requirements, please see *PLOS ONE’s* [submission guidelines](https://journals.plos.org/plosone/s/submission-guidelines), [formatting template](https://journals.plos.org/plosone/s/file?id=wjVg/PLOSOne_formatting_sample_main_body.pdf), and [figure guidelines](https://journals.plos.org/plosone/s/figures). If you have any questions, please contact us at plosone@plos.org.

7. To ensure your figures meet our technical requirements, please run each figure included in your submission files through the PACE tool: [https://eur01.safelinks.protection.outlook.com/?url=https%3A%2F%2Fpacev2.apexcovantage.com%2F&data=05%7C02%7Cylva.persson%40sva.se%7C794a351996ed4856452a08dc328b1d42%7Cb64756aa7df04f2ea41171214a50af24%7C0%7C1%7C638440822087760297%7CUnknown%7CTWFpbGZsb3d8eyJWIjoiMC4wLjAwMDAiLCJQIjoiV2luMzIiLCJBTiI6Ik1haWwiLCJXVCI6Mn0%3D%7C0%7C%7C%7C&sdata=LqG5u9sGmkvNR49HZtdcK12uAO4djwvPvnWK8gwLEmY%3D&reserved=0](https://pacev2.apexcovantage.com/). PACE will assess whether your figures meet our technical requirements and will fix the figure(s) or identify any problem(s) that cannot be automatically fixed. It can also convert figures to TIFF format, resize, and rename figures to meet our naming conventions.
To use PACE, first register as a user. Follow the instructions on the site for assessing and converting your figure files. If you experience any difficulty using this tool or have questions about any of the figures and/or images in your paper, please inform the journal office in your response letter.

**Response**

The figure we used in this manuscript has been checked using PACE tool and will be uploaded during submission of the amended version.

The PACE modifications are as follows:

- Image Problems: No problems were mentioned
- PACE Adjustments: Dimensions are adjusted to 7.5in W x 3in H and JPG file is converted to a valid TIF file.
- Results: Please inspect this version for image clarity and content. We checked it.
- Upload Date: 02/21/2024 03:25 AM
